# A scalable, knowledge-based analysis framework for genetic association studies

**DOI:** 10.1186/1471-2105-14-312

**Published:** 2013-10-23

**Authors:** James W Baurley, David V Conti

**Affiliations:** 1 Bioinformatics Research Group, Bina Nusantara University, Jakarta, Indonesia; 2 BioRealm LLC, Monument, USA; 3 Department of Preventive Medicine, University of Southern California, Los Angeles, USA

## Abstract

**Background:**

Testing for marginal associations between numerous genetic variants and disease may miss complex relationships among variables (e.g., gene-gene interactions). Bayesian approaches can model multiple variables together and offer advantages over conventional model building strategies, including using existing biological evidence as modeling priors and acknowledging that many models may fit the data well. With many candidate variables, Bayesian approaches to variable selection rely on algorithms to approximate the posterior distribution of models, such as Markov-Chain Monte Carlo (MCMC). Unfortunately, MCMC is difficult to parallelize and requires many iterations to adequately sample the posterior. We introduce a scalable algorithm called PEAK that improves the efficiency of MCMC by dividing a large set of variables into related groups using a rooted graph that resembles a mountain peak. Our algorithm takes advantage of parallel computing and existing biological databases when available.

**Results:**

By using graphs to manage a model space with more than 500,000 candidate variables, we were able to improve MCMC efficiency and uncover the true simulated causal variables, including a gene-gene interaction. We applied PEAK to a case-control study of childhood asthma with 2,521 genetic variants. We used an informative graph for oxidative stress derived from Gene Ontology and identified several variants in *ERBB4*, *OXR1*, and *BCL2* with strong evidence for associations with childhood asthma.

**Conclusions:**

We introduced an extremely flexible analysis framework capable of efficiently performing Bayesian variable selection on many candidate variables. The PEAK algorithm can be provided with an informative graph, which can be advantageous when considering gene-gene interactions, or a symmetric graph, which simply divides the model space into manageable regions. The PEAK framework is compatible with various model forms, allowing for the algorithm to be configured for different study designs and applications, such as pathway or rare-variant analyses, by simple modifications to the model likelihood and proposal functions.

## Background

Complex biological pathways play a role in many common diseases, such as heart disease and cancer. Genetic variants in the genes involved in these pathways may independently or in combination influence disease risk. The majority of genetic association studies, however, report results from sequentially testing marginal associations between each genetic variant and disease. While this approach has certainly had many successes, it is unlikely to capture many relationships among variables, such as gene-gene interactions [1,2].

For applications with few candidate genetic variants, conventional multivariable regression modeling works well. Here, the analyst uses a combination of model fitting and an understanding of biological context to build a model that may include confounding and interaction variables. When there are many variables, however, the analyst must turn towards automated variable selection algorithms, such as stepwise regression. These approaches often result in a single best model that ignores the uncertainty in the decisions made in building it.

Bayesian approaches to variable selection address the uncertainty issue directly by using the posterior distribution of models rather than a single best model for inference [3]. When there are a small number of variables, exact computation can be accomplished by enumerating all possible models. With many variables this quickly becomes intractable and the posterior must be approximated with Markov Chain Monte Carlo (MCMC) methods. Recently, Bayesian frameworks have been introduced for modeling complex interactions [4,5] and risk scores for rare genetic variants [6]. These MCMC approaches, however, have been limited to applications with a relative small number of candidate variables because they do not efficiently sample the posterior distribution.

We introduce a framework called PEAK that improves the efficiency of MCMC by dividing a large set of variables into related groups using a rooted graph that resembles a mountain peak. Our algorithm is flexible to different model specifications and takes advantage of parallel computing and existing biological databases when available. The framework will allow for comprehensive analyses of genetic association studies using modern Bayesian modeling approaches.

## Methods

The PEAK framework is an implementation of Bayesian variable selection for applications with many candidate variables (e.g., genetic variants). Inference is based on the posterior distribution of models. The posterior probability for model *M* is given by

$$p(M|D)=\frac{p(D|M)p(M)}{\sum_{M\in M}p(D|M)p(M)}$$

where **D** is the observed data, *p*(**D**|*M*) is the marginal likelihood for model *M* (integrating over any parameters in that model), *p*(*M*) is the prior for the particular model (if specified), and the denominator is a constant found by summing over all models **M**. The marginal posterior probability for any variable of interest (e.g., genetic or environmental risk factors or interactions) is computed by summing the probabilities for each model containing the variable,

$$p(I_{p}=1|D)=\underset{M\in M}{\sum }p(M|D)I_{p\in m}$$

where *I*_*p*∈*m*_ is an indicator if the variable *p* is in the model *m*. Additionally, the Bayes factor (BF), the ratio of posterior to prior odds, is used to evaluate the extent the data supports a particular variable,

$$\text{BF}=\frac{p(I_{p}=1|D)/(1-p(I_{p}=1|D))}{p(I_{p}=1)/(1-p(I_{p}=1))}$$

where a Bayes factor of 1–3 is considered weak evidence, 3–20 positive evidence, 20–150 strong evidence, and greater than 150 very strong evidence [7].

In many applications, an exhaustive search of **M** is intractable and the posterior distribution is approximated using MCMC methods. The PEAK framework is designed to efficiently sample from *p*(**M**|**D**) using MCMC by using a graph to divide the set of candidate variables into groups.

### Model specification

The form of models considered by PEAK is flexible, and the model likelihood is specified by the user. In this implementation, we fit generalized linear models (GLM). The data **D** contain the outcome variable *Y* and a matrix of *P* explanatory variables **X** (which may include pairwise and higher-order interaction variables). The expected value of *Y*_*i*_, the outcome variable for individual *i*, depends on the linear predictors through the link function *g* such that,

$$g(\mu_{i})=\beta_{0}+\overset{P}{\underset{p}{\sum }}\beta_{p}X_{\text{ip}}I_{p}$$

where *μ*_*i*_=E(*Y*_*i*_),*β*_*p*_ is the regression coefficient of variable *p*, and *I*_*p*_ is a variable indicating if *X*_*p*_ is included in the model *M*. The desired link function, the highest order interactions to consider in a model (none, pairwise, three-way, etc.) and **D** are provided by the user.

### Graph-based Metropolis-Hastings

**Figure 1:**
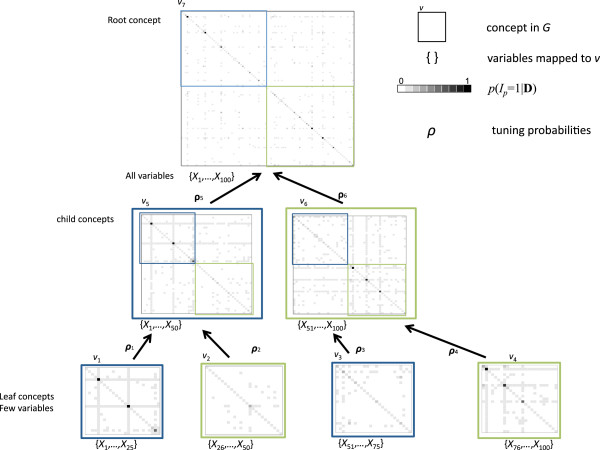
**Managing the model space with a directed acyclic graph.** This graph contains seven concepts (shown as boxes). All variables are mapped to the root concept *v*_7_. Smaller sets of variables are mapped to child concepts (vertices towards the bottom of the graph). A small portion of the model space is explored for the set of variables mapped to that concept (displayed in brackets). Arrows indicate the direction of graph communications, with probabilities *ρ* customizing the Metropolis-Hastings proposal density. For each concept, the posterior estimates are shown for main effect variables (diagonals) and pair-wise interactions (off-diagonals). Darker shades indicate higher posterior probabilities. The boxes (blue and green) within a matrix denotes the portion of the model space previously searched.

The PEAK framework implements a random-walk Metropolis-Hastings (M-H) algorithm [8] with a custom proposal density. The proposal is customized through a vector of tuning probabilities ***ρ***. If the tuning probabilities were equal for all variables, then the PEAK algorithm reduces to traditional M-H. PEAK customized the proposal using a graph to break the model search space down into local regions (Figure 1).

The *P* candidate variables are mapped to concepts, which are related through a directed acyclic graph (DAG). We specify a rooted DAG *G*=(*V*,*E*) consisting of a set of vertices *V* (concepts) and a set of directed edges *E* that connect pairs of concepts. Concepts represent groups of variables (e.g., SNPs that are within a gene). The edges may represent different relationships and can be either generic (e.g., *part-of*, *is-a*) or domain specific (e.g., a gene *regulates*). *G* has a root vertex (the peak) in which all other vertices and edges are oriented, with vertices closer to the root being parents and those further away being children.

The algorithm begins by estimating the posterior probabilities for the set of variables mapped to the leaves of *G*. As concepts join in *G*, the algorithm estimates the posterior probabilities for a larger set of variables, returning to regions identified by local searches performed earlier by the tuning probabilities (see Algorithm 1).

At the leaves of *G*, the tuning probabilities ***ρ*** are user defined. Since we are interested in models with interactions, we set the default for these tuning probabilities so the proposed model *M*^′^, on average, involves two explanatory variables. For the internal vertices, ***ρ*** is weighted to ensure that the entire model space can be explored (i.e., no variables are always (*ρ*=1) or never (*ρ*=0) proposed in the new model *M*^′^). To accomplish this, we place a *beta* prior on the tuning probabilities to shrink the posterior probabilities computed at the end of Algorithm 1 for vertex *v*’s children towards the default tuning probabilities for *v*.

**Algorithm 1** Estimation of the posterior probabilities for all variables mapped to concept *v* in the directed acyclic graph *G*

### Job management and parallel computing

The PEAK software queues Algorithm 1 based on the graph using Portable Batch System (PBS). Initially all the leaves of *G* are queued and executed in parallel up to the number of processors available. For internal vertices, a job is queued immediately after the completion of its children and executed when a processor becomes available. Algorithm 1 is currently implemented in [R] [9].

### Simulations

We used data simulations to compare the performance of the PEAK algorithm, with different graphs and variable mappings, to the standard M-H algorithm. For the simulations, we assumed a binary outcome *Y*_*i*_ where *Y*_*i*_∼Bernoulli(*π*_*i*_) and a link function, $g(\pi_{i})=\log\left(\frac{\pi_{i}}{1-\pi_{i}}\right)$. Each data replicate included *J*=1,000 binary variables, which we refer to as genetic variants, and J2=499,500 interaction variables. The outcome variable *Y* was generated under additive and interaction true models, where variants *X*_7_ and *X*_10_ were involved: 

•**Scenario 1:***g*(*π*_*i*_)=*β*_0_+*β*_7_*X*_7,*i*_+*β*_10_*X*_10,*i*_ with *β*_7_ and *β*_10_= log(1.5)

•**Scenario 2:**$g(\pi_{i})=\beta_{0}+\beta_{7}X_{7,i}+\beta_{10}X_{10,i}+\beta_{X_{7}:X_{10}}X_{7,i}:X_{10,i}$ with no simulated main effects (*β*_7_ and *β*_10_= log(1.0)) and an interaction effect between *X*_7_ and *X*_10_ ($\beta_{X_{7}:X_{10}}=\log(3.0)$)

For each scenario, we generated ten data replicates of 1,000 individuals for analysis.

**Figure 2:**
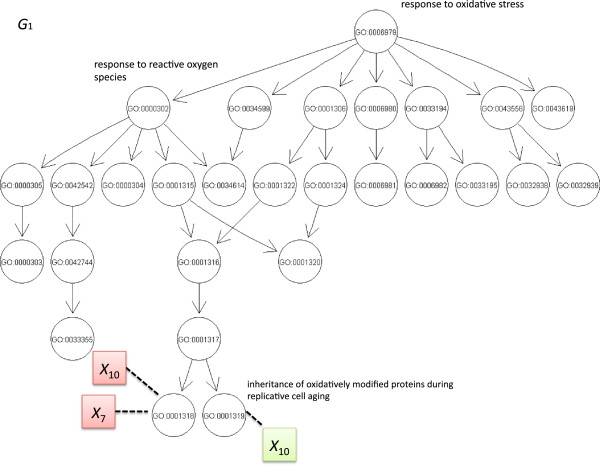
**Informative graph *G***_**1**_** extracted from Gene Ontology:*response to oxidative stress*.***G*_1_ was extracted from Gene Ontology and contains 28 concepts (circles with the GO identifier). Concepts become more general towards the top of the graph and thus have larger sets of variables. The simulated causal variants *X*_7_ and *X*_10_ were mapped to a common concept (GO:0001318) and then sibling concepts (GO:0001318 and GO:0001319). Non-causal variants were randomly distributed across the graph.

**Figure 3:**
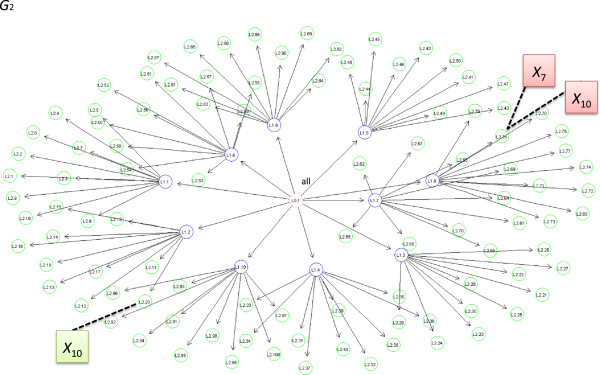
**Symmetric graph *G***_**2**_**.***G*_2_ is a symmetric tree, with 100 leaves (green), 10 intermediate vertices (blue), and one root. The simulated genetic variants were distributed across the green nodes. The causal variants were first mapped to the same concept and then separated to distant concepts.

As input to the PEAK algorithm, we generated two different graphs. The first graph was obtained from the Gene Ontology database for the biological process “*response to oxidative stress*”. This six-level informative graph is denoted *G*_1_ and is presented in Figure 2. *G*_1_ was used in the analysis of the simulation datasets and a genome-wide study of childhood asthma. The second graph was not derived from a biological database. This graph (denoted *G*_2_) was symmetric with many concepts joining in three levels (see Figure 3). The causal variants *X*_7_ and *X*_10_ were mapped in two different ways to these graphs. For the informative graph, these variants were mapped to the same concept (term GO: 0001318) and then to separate concepts (terms GO: 0001318 and GO:0001219) sharing a common biological process (i.e. siblings in the graph - see Figure 2). For the symmetric graph, the causal variants were mapped to the same concept and then concepts with a distant common ancestor (see Figure 3). The non-causal variants were evenly mapped to the leaves of *G*.

The M-H and PEAK algorithms were configured to consider models with any number of variables, including pairwise interactions, and run on all the Scenario 1 and 2 datasets. The M-H algorithm was run for *u*=800,000 iterations. The PEAK algorithm was configured for *G*_1_ and *G*_2_ and the different variable mappings. Algorithm 1 was run for *u*=100,000 iterations for all vertices below the root and *u*=300,000 iterations for the root. Multiple chains with different initial values were used to evaluate convergence. For comparison to traditional approaches, the best model was chosen by Bayesian information criterion (BIC) using forward stepwise logistic regression on each dataset.

## Results

### Statistical inference

**Figure 4:**
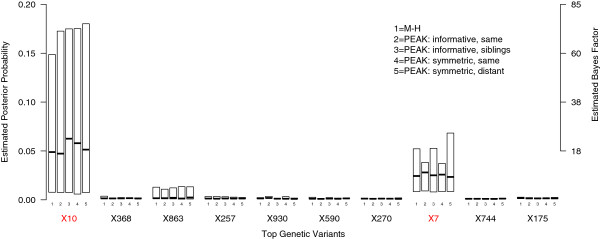
**Estimated marginal posterior probabilities for Scenario 1 datasets.** The top estimated posterior probabilities were summarized across the Scenario 1 datasets. The box plots confirms that PEAK was converging to the same estimates as the M-H algorithm, regardless of the graph used and the variable mappings. The simulated causal variants *X*_10_ and *X*_7_ had elevated posterior probabilities and evidence of association across datasets, while the non-causal variants did not. Note that the whiskers and outliers were not drawn on the box plots.

**Figure 5:**
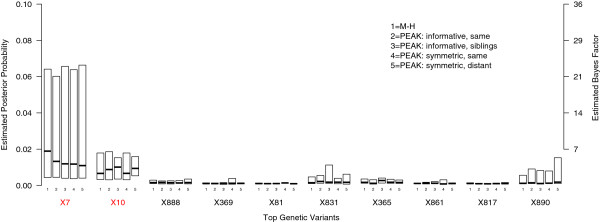
**Estimated marginal posterior probabilities for Scenario 2 datasets.** The top estimated posterior probabilities were summarized across the Scenario 2 datasets. The posterior estimates were very similar across algorithms, indicating convergence. Marginally, *X*_10_ had low posterior estimates, meaning it was infrequently included in the model without the interaction with *X*_7_. Note that the whiskers and outliers were not drawn on the box plots.

The marginal posterior probabilities for each genetic variant were averaged over the data replicates. The top ten genetic variants obtained from the M-H algorithm were then compared to PEAK. While the posterior estimates varied by data set as expected, the distribution of posterior estimates were highly consistent across the M-H and PEAK algorithms (see box plots in Figures 4 and 5 for Scenario 1 and 2 datasets respectively). Thus, under the configurations used in these analyses, inference using the PEAK algorithm was equivalent to the traditional M-H algorithm.

For the Scenario 1 datasets, the simulated causal variants *X*_10_ and *X*_7_ were among the top variants. While some non-causal variants had elevated posterior probabilities in individual datasets (e.g., *X*_368_), only *X*_10_ and *X*_7_ showed evidence across datasets. The maximum posterior probability for *X*_10_ was 0.63, meaning that for this dataset, there was very strong evidence in favor of including this variable in the model (Bayes factor of 570). Among the other Scenario 1 datasets, there was positive evidence for including *X*_10_, but with much lower posterior probabilities (Figure 4 - median posterior: 0.05, Bayes factor: 17). The maximum posterior estimate for *X*_7_ was 0.07 and had strong evidence of association (Bayes factor: 26). Across datasets, there was positive evidence for including this variant in the model (median posterior: 0.02, Bayes factor: 8). No pairwise interactions had elevated posterior probabilities. This was expected given the data was simulated under an additive model. Using forward stepwise regression on the Scenario 1 datasets without considering interaction variables, *X*_10_ was included in the best model six times and *X*_7_ was included four times.

For the Scenario 2 datasets, the simulated causal variants were again among the top variants. Marginally, *X*_7_ showed evidence across the datasets (maximum posterior: 0.97, median: 0.02), whereas *X*_10_ had a rather low posterior overall (see Figure 5, median: 0.007). This reflects that *X*_10_ was infrequently included without the interaction variable between *X*_7_ and *X*_10_. This interaction had extremely strong evidence of association in one dataset (maximum posterior: 0.96), and positive evidence in the others (median posterior: 0.001). When analyzed these datasets using forward stepwise regression, *X*_7_ was included in the best model three times, and *X*_10_ was never included in the best model, indicating that *X*_10_ lacked a marginal effect in these datasets.

### Computational aspects

**Figure 6:**
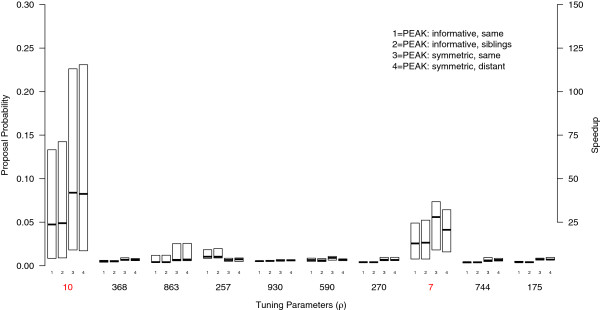
**Tuning probabilities and speedup for Scenario 1 datasets.** The proposal probabilities used in Algorithm 1 for the root were summarized for the Scenario 1 datasets. Here, the symmetric graph outperformed the informative graph. All of the PEAK algorithm configurations offered considerable speedup over the M-H algorithm (values shown on right axis). Note that the whiskers and outliers were not drawn on the box plots.

**Figure 7:**
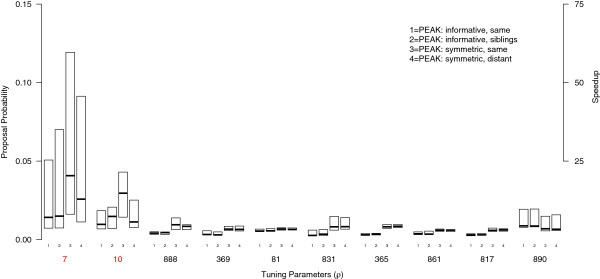
**Tuning probabilities and speedup for Scenario 2 datasets.** The proposal probabilities used in Algorithm 1 for the root were summarized for the Scenario 2 datasets. With the true model containing an interaction between *X*_7_ and *X*_10_, the performance was dependent on both the graph used and where the variables were mapped to the graph. The symmetric graph with the causal variants mapped to the same concept had the best performance. Note that the whiskers and outliers were not drawn on the box plots.

The PEAK algorithm selects variables to include in the proposed model based on a vector of tuning probabilities ***ρ***. Unlike the standard M-H algorithm, the probability of including each variable is dynamic and may change as a function of the evidence from lower levels in the graph. This can result in each variable having different probabilities of being included in the proposed model. The number of iterations (time) expected to propose the causal variant is proportional to the tuning probabilities, which are influenced by the graph and the way the variables are mapped to the graph. We defined speedup as the ratio of the custom tuning probabilities (i.e. *ρ*) used at the root of *G* to the uniform proposal probabilities used in the M-H algorithm. The tuning probabilities were summarized for the top genetic variants and compared to the M-H algorithm (Figures 6 and 7 respectively).

For the Scenario 1 datasets, the tuning probabilities for the non-causal variants were close to the default of 0.002, implying they were proposed with relatively low frequency. The causal variants had elevated tuning probabilities across the datasets (see Figure 6). For example, with the informative graph *G*_1_, the median probability for *ρ*_10_ was 0.05 representing a speedup over the M-H algorithm of approximately 25. And for the symmetric graph *G*_2_, the median probability for *ρ*_10_ was 0.08 with a speedup of 40. This implies that the PEAK algorithm proposed the causal variants more often, and in expectation, greatly reduced the number of iterations (time) needed to propose the true model. For both causal variants, the tuning probabilities were higher for the symmetric graph than the informative graph (see Figure 6). Thus, having fewer variants mapped per leaf may have yielded a slight advantage to the symmetric graph. Under an additive true model, the differences in variable mappings did not appear to significantly influence the tuning probabilities. There was a slight decrease in *ρ*_7_, however, when the causal variants where mapped to distant concepts in the symmetric graph.

For the Scenario 2 datasets, PEAK again improved the rate of convergence (see Figure 7). With the true model containing an interaction, however, the performance was more dependent on the graph and where the causal variables were mapped to the graph. Overall, the symmetric graph had higher values of *ρ*_7_ than the informative graph. The values of *ρ*_10_ were similar for the informative graph regardless to whether *X*_10_ shared the same concept or parents concepts with *X*_7_. For the symmetric graph, when *X*_10_ was mapped far away from *X*_7_, the values of *ρ*_10_ decreased, implying that both *X*_10_ and the interaction with *X*_7_ would be proposed less frequently in this case. The results show that for these data, the symmetric graph with the causal variables mapped to the same concept would be expected to converge the fastest.

The speedup from using parallel computing is highly dependent on the graph used. Using 100 computing nodes for the processing of the symmetric graph, the speedup was 13.9. For the informative graph using 12 computing nodes, the speedup was only 1.2.

### Application to a Genome-wide association study of childhood asthma

Asthma is the most common chronic disease in children. There is evidence that cellular responses to oxidative stress are important in the development and progression of asthma [10,11]. Variants in genes involved in this biological process may independently and jointly influence asthma risk. Using data from a genome-wide association study (GWAS) of childhood asthma and Gene Ontology, PEAK was used to find associations between 2,521 variants in oxidative stress genes and childhood asthma.

The Children’s Health Study (CHS) is an ongoing cohort study spanning 16 southern California communities investigating both genetic and environmental factors related to childhood asthma and lung function growth [12]. The CHS GWAS was a nested case-control sample selected from the CHS cohorts genotyped for over 500,000 single-nucleotide polymorphisms (SNPs). After quality control screening, a total of 3,000 subjects (1,249 cases, 1,751 controls) were available for analysis. Genotype imputation was performed using MACH [13] with the HapMap release 21 haplotypes as a reference. We extracted 168 genes associated with the concept “*response to oxidative stress*” in Gene Ontology (source date: 20100320). The UCSC hg19 start and stop position for each gene were extended by 5 kilobases and converted to compatible coordinates using liftOver. For these regions, 2,521 genotyped SNPs with an imputation quality of *r*^2^≥0.3 and minor allele frequency ≥0.01 were candidate variables. Logistic regression models were considered, with imputation dosages of the minor allele being used for each SNP, and including covariates to adjust for sex, CHS cohort, self-identified ethnicity, and ancestry covariates obtained from the software STRUCTURE [14]. Over 3 million interaction variables were considered with no restriction on the size of the model. An extended version of the *G*_1_ graph was used with the 168 genes linked to the 35 Gene Ontology concepts. Algorithm 1 was run for 100,000 iterations for concepts below the root and one million iterations for the root. The root process took 62.5 hours on an AMD Opteron 2.3 GHz.

A summary of the top SNPs associated with childhood asthma are given in Table 1. The variant with the most evidence of association with asthma was rs13008370 in the *ERBB4* gene on chromosome 2 (Posterior probability 47%, Bayes Factor: 157). Other SNPs within *ERBB4* were associated with asthma included rs11680307 (Bayes Factor: 42), rs1521658 (Bayes Factor: 26), and rs6435692 (Bayes Factor: 4). Another region of interest was *BCL2* on chromosome 18 flagged by rs2156192 (Bayes Factor: 72), rs9972996 (Bayes Factor: 17), and rs2551402 (Bayes Factor: 6). Both *ERBB4* and *BCL2* were linked to *response to hydrogen peroxide* in GO. The top interaction involved rs2156192 in *BCL2* and rs10305724 in *ARNT* on chromosome 1 (Posterior probability: 0.042, Bayes Factor: 414). Other interactions were found but with estimated posterior probabilities <0.01, many of which included either *BCL2* or *ERBB4*.

**Table 1:** Top marginal posterior probabilities and Bayes factors for the childhood asthma application

**SNP**	**Gene**	**Posterior estimate**	**Bayes factor**
rs13008370	*ERBB4*	0.47	157
rs2156192	*BCL2*	0.29	72
rs11680307	*ERBB4*	0.19	42
rs1521658	*ERBB4*	0.13	26
rs10108813	*OXR1*	0.10	20
rs9972996	*BCL2*	0.09	17
rs10305724	*ARNT*	0.04	8
rs3793371	*NAPRT1*	0.03	6
rs2551402	*BCL2*	0.03	6
rs1954752	*OXR1*	0.03	6
rs3019308	*OXR1*	0.03	5
rs12950972	*CYGB*	0.03	5
rs1983298	*PTPRN*	0.02	4
rs6435692	*ERBB4*	0.02	4
rs1574311	*TPM1*	0.02	3
rs2687975	*LIAS*	0.02	3
rs3788310	*TXNRD2*	0.02	3
rs4647519	*FANCC*	0.02	3
rs1050255	*TPM1*	0.02	3

2,521 SNPs genotyped in a GWAS of childhood asthma were extracted from 168 genes mapped to *response to oxidative stress* in Gene Ontology. The top estimated marginal posterior probabilities and Bayes factors are reported.

## Discussion

The PEAK algorithms can be provided with different types of graphs. Informative graphs group variables conceptually. These graphs can be created by the user or automatically extracted from existing databases, such as Kyoto Encyclopedia of Genes and Genomes (KEGG) pathways [15] or Gene Ontology. A hypothesized disease pathway, for example, can be captured by *G* with genetic variants or environmental factors being mapped to steps within the pathway. Informative graphs allows inference on any user-defined functional unit that exists within the graph, for example genes or regions, steps in biological processes, or pathways within a larger network. These graphs may have an uneven distribution of variables mapped across concepts in the graph. If this unbalance is too extreme, there are too many variables with no information to customize the proposal. In this case, we recommend merging concepts or connecting additional concepts to widening the base of the graph.

There are applications where annotation does not exists or the knowledge captured is too sparse or vague to group variables in a meaningful way. In cases with no information, we recommend a symmetric graph with the set of variables divided into groups containing up to 50 variables. While the performance of our method is sensitive to graphs or variable mappings that do not accurately represent biological truth, there is still a benefit in dividing a large set of variables using a graph. In our simulations, we showed that a symmetric graph had better performance than the M-H algorithm because it allowed many small portions of the model space to be considered in parallel. The efficiency, however, may be dictated by the marginal effects of a variable, which may be small or non-detectable for certain types of interactions. For example, we had trouble finding the true interaction when the variants in Scenario 2 were mapped to distant relatives in *G*_2_. When variables involved in a true interaction are mapped to the same or closely related concept (as in *G*_1_), they are discovered near the bottom of the graph and these findings are propagated up the graph.

The PEAK framework improves performance of the M-H algorithm by constructing a custom proposal density that can quickly explore the model space tagged by earlier searches. There is a tradeoff, however, between exploring the entire model space and discounting regions as uninteresting. The former converges to the same posterior distribution of models as direct computation (exhaustively visiting all models), while the latter is necessary for the algorithm to complete in a reasonable amount of time. While not in a MCMC framework, other Bayesian variable selection algorithms have summarized the posterior distribution of models for a subset of models defined using a search heuristic such as Occam’s window [16] or the leaps and bounds algorithm [17]. The PEAK algorithm approximates the posterior distribution of models of interest, but given enough iterations, as shown in our simulations, approximates the posterior distribution of all models. The PEAK algorithm is not a traditional adaptive algorithm because the proposal is customized by the Metropolis-Hastings algorithms that ran on child vertices of *G*. The target distribution is not biased since the tuning probabilities are set before the Metropolis-Hastings algorithm begins and the proposal is not adapting while the chain is executing.

The PEAK framework is capable of scaling to high-throughput genotyping and sequencing applications (e.g., rare variants analysis and gene-gene interaction scans). Although large applications would require considerable computing resources, cluster and cloud computing are becoming inexpensive and accessible. For smaller applications (e.g., candidate gene studies), PEAK could be run on a workstation with a multicore processor.

## Conclusions

We have introduced a flexible analysis framework capable of efficiently performing Bayesian variable selection in data with many candidate variables. The PEAK framework manages an extremely large model space by grouping variables on a graph and using many local searches to construct a custom proposal density for the Metropolis-Hastings algorithm. The PEAK algorithm can be provided with an informative graph, which can be advantageous when considering gene-gene interactions, as demonstrated in the asthma application. Alternatively, PEAK may be provided with a symmetric graph, which simply divides the model space into manageable regions. The PEAK framework is compatible with various model forms by modifications to the proposal and model likelihood functions, allowing the algorithm to be configured for different study designs and applications, such as family-based studies and rare-variant analysis of sequencing data.

## Abbreviations

MCMC: Markov chain Monte Carlo; GLM: Generalized linear model; M-H: Metropolis-Hastings; DAG: Directed acyclic graph; SNP: Single nucleotide polymorphism; GO: Gene ontology; CHS: Children’s health study.

## Competing interests

Dr. Baurley is co-founder and an employee of BioRealm LLC.

## Authors’ contributions

Development of the method (JWB & DVC); Developing the software (JWB); Writing the manuscript (JWB & DVC). Both authors read and approved the final manuscript.
